# Supplementary material for: Community-wide deworming strategies to reduce high hookworm burden in endemic communities: Results from a cluster randomized trial in Southern India
Source: PLoS Negl Trop Dis. 2026 Apr 16;20(4):e0013440. doi: 10.1371/journal.pntd.0013440 (PMC13138741; doi:10.1371/journal.pntd.0013440)
Supplement: S1 Table — * 95% CI adjusted for clustering at the village level. ¶ EPG (eggs per gram of faeces) counted by the McMaster technique; SE-standard error adjusted for clustering at the village level. (DOCX) [file pntd.0013440.s001.docx]

***S1 Table.* Age-specific (baseline) prevalence and intensity of hookworm infection**

| **Age group** | **Number sampled** | **Percentage prevalence (95% CI)*** | **Mean (SE) EPG^¶^** |
| --- | --- | --- | --- |
| <=4 years | 131 | 8.4 (4.7-14.5) | 74 (42) |
| 5-9 years | 363 | 12.4 (8.5-17.7) | 43 (16) |
| 10-14 years | 279 | 16.1 (11.9-21.5) | 62 (21) |
| 15-24 years | 272 | 18.4 (12.8-25.7) | 159 (61) |
| 25-34 years | 405 | 20.5 (16.9-24.6) | 95 (18) |
| 35-44 years | 320 | 19.1 (15.6-23.1) | 151 (48) |
| 45-54 years | 223 | 30.5 (23.9-38.0) | 310 (113) |
| >=55 years | 89 | 25.8 (19.0-34.1) | 219 (96) |
| Overall | 2082 | 18.5 (16.2-21.2) | 125 (26) |
| * 95% CI adjusted for clustering at the village level | | | |
| ^¶^ EPG (eggs per gram of faeces) counted by the McMaster technique; SE-standard error adjusted for clustering at the village level | | | |
