# Supplementary material for: Community-wide deworming strategies to reduce high hookworm burden in endemic communities: Results from a cluster randomized trial in Southern India
Source: PLoS Negl Trop Dis. 2026 Apr 16;20(4):e0013440. doi: 10.1371/journal.pntd.0013440 (PMC13138741; doi:10.1371/journal.pntd.0013440)
Supplement: S2 Table — * 95% CI for ORs adjusted for clustering at the village level. ¶ Reference category: age group <5 years. § Reference category: SES category- low. (DOCX) [file pntd.0013440.s002.docx]

***S2 Table.* Results of logistic regression analysis to explore the factors associated with hookworm infection at baseline**

| **Variable** | **Univariate logistic regression** | | **Multivariable logistic regression** | |
| --- | --- | --- | --- | --- |
|  | **Odds ratio (95% CI)*** | ***P*-value** | **Odds ratio (95% CI)*** | ***P*-value** |
| Age group^¶^ | | | | |
| 5-9 years | 1.54 (0.78-3.04) | 0.209 | 1.65 (0.82-3.33) | 0.161 |
| 10-14 years | 2.10 (1.13-3.89) | 0.019 | 2.38 (1.22-4.64) | **0.011** |
| 15-24 years | 2.46 (1.22-4.96) | 0.012 | 2.92 (1.35-6.31) | **0.007** |
| 25-34 years | 2.81 (1.50-5.26) | 0.001 | 3.42 (1.69-694) | **0.001** |
| 35-44 years | 2.57 (1.34-4.91) | 0.004 | 2.95 (1.42-6.13) | **0.004** |
| 45-54 years | 4.79 (2.57-8.91) | <0.001 | 5.26 (2.61-10.58) | **<0.001** |
| >=55 years | 3.80 (1.84-7.85) | <0.001 | 4.31 (1.91-9.74) | **<0.001** |
| Male gender | 1.25 (0.98-1.61) | 0.076 | 1.31 (1.02-1.67) | **0.032** |
| SES category^§^ | | | | |
| Middle | 1.35 (0.99-1.84) | 0.057 | 1.22 (0.88-1.68) | 0.229 |
| High | 0.90 (0.59-1.35) | 0.600 | 0.89 (0.59-1.33) | 0.557 |
| Mud flooring | 1.39 (1.08-1.78) | 0.010 | 1.31 (1.02-1.68) | **0.038** |
| Drinking purified water | 0.73 (0.55-0.95) | 0.022 | 0.75 (0.56-0.99) | **0.049** |
| Regular footwear usage | 0.81 (0.59-1.11) | 0.187 | 0.84 (0.60-1.18) | 0.322 |
| Washing hands with soap and water after defecation | 0.97 (0.66-1.43) | 0.881 | 1.14 (0.71-1.82) | 0.594 |
| Performing agriculture-related activities in the preceding 6 months | 1.43 (0.97-2.11) | 0.068 | 0.83 (0.52-1.31) | 0.420 |
| Regular contact with animals | 1.40 (1.03-1.90) | 0.030 | 1.06 (0.76-1.49) | 0.726 |
| * 95% CI for ORs adjusted for clustering at the village level | | | | |
| ^¶^ Reference category: age group <5 years  ^§^ Reference category: SES category - low | | | | |
