# Supplementary figures and images for: Community-wide deworming strategies to reduce high hookworm burden in endemic communities: Results from a cluster randomized trial in Southern India
Source: PLoS Negl Trop Dis. 2026 Apr 16;20(4):e0013440. doi: 10.1371/journal.pntd.0013440 (PMC13138741; doi:10.1371/journal.pntd.0013440)

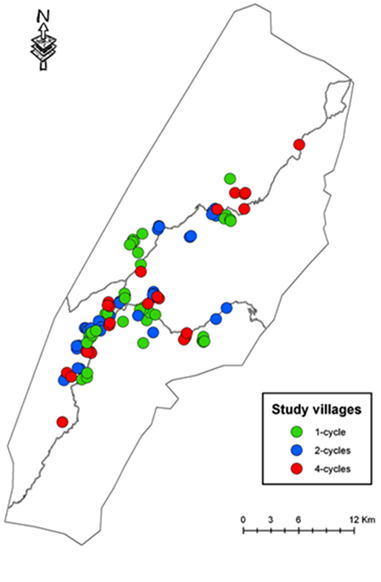

Supplement: S1 Fig — The map was created entirely from self-collected primary data for this study. The area boundaries, as well as all point locations, were digitized by the study team based on our own field data. No external basemap tiles, satellite images, proprietary map services (e.g., Google Maps, MapQuest), or third‑party shapefiles (such as Natural Earth, OpenStreetMap, or other providers) were used to generate the underlying boundary layer shown in S1 Fig. (TIF) [file pntd.0013440.s003.tif]

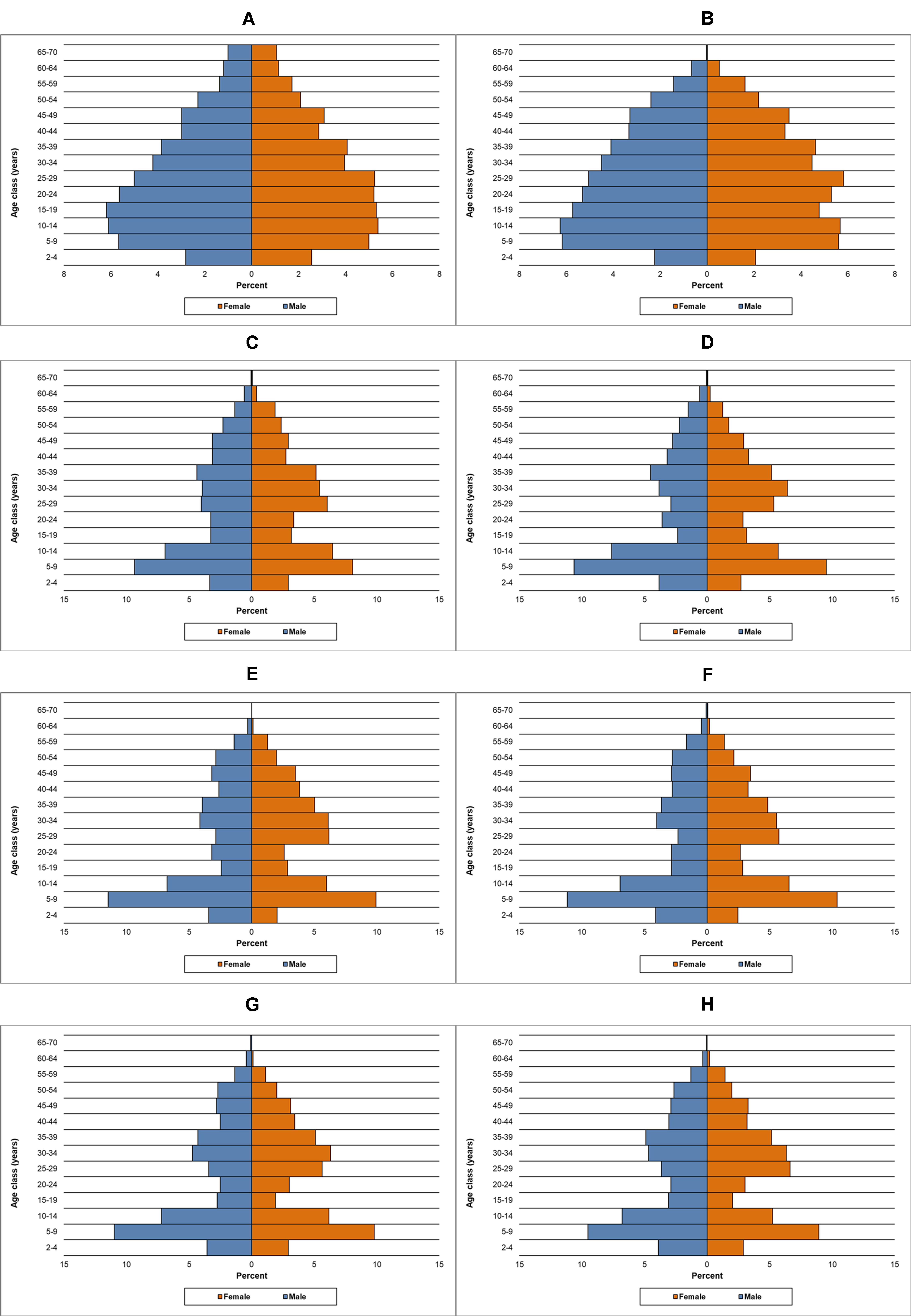

Supplement: S2 Fig — (TIF) [file pntd.0013440.s004.tif]
