# Supplementary material for: Community-wide deworming strategies to reduce high hookworm burden in endemic communities: Results from a cluster randomized trial in Southern India
Source: PLoS Negl Trop Dis. 2026 Apr 16;20(4):e0013440. doi: 10.1371/journal.pntd.0013440 (PMC13138741; doi:10.1371/journal.pntd.0013440)
Supplement: Attachment — Submitted filename: 20260204-STH-Alb-Paper-Response-Reviewer-Comments.docx [file pntd.0013440.s006.docx]

**RESPONSE TO REVIEWER AND EDITOR COMMENTS**

***PNTD-D-25-01140:*** *Community-wide deworming strategies to reduce high hookworm burden in endemic communities: Results from a cluster randomized trial in southern India*

*We thank the reviewer and the editor for their thorough and helpful review of this manuscript. We have responded to the comments and provide below their original comments followed by our responses (in italics).*

**Reviewer 1:**

1. Overarching question – Given the emphasis on achieving and sustaining the WHO target for STH elimination as a public health problem (<2% prevalence of moderate-to-heavy intensity infections, line 418-419), it seems the study villages met that criteria at the onset of the study (line 262-263; <1.2% moderate to heavy intensity infections). How do you think that baseline condition affects the generalizability of your work to higher intensity communities?

**RESPONSE:** *We appreciate the reviewer’s observation that the baseline prevalence of moderate-to-heavy intensity (MHI) infections in the study villages (<1.2%) was already below the WHO threshold for elimination as a public health problem. The WHO target of <2% prevalence of MHI infections is aimed at morbidity control rather than transmission interruption; achieving transmission interruption will require post-treatment prevalence of ≤2% (Truscott et al. Parasit Vectors. 2017: 321). Modelling studies indicate that targeted deworming of pre-school and school-aged children alone may not achieve this, particularly in hookworm-endemic areas with untreated adult reservoirs of infection (Anderson et al. PLoS Negl Trop Dis. 2015; 9: e0003897). Moreover, reinfection after effective treatment remains a challenge (Jia et al. PLoS Negl Trop Dis. 2012; 6: e1621), requiring deworming to be continued indefinitely in absence of substantial improvements in water, sanitation and hygiene (WASH). Hence, alternative deworming strategies, especially in low-intensity settings, need to be explore the possibility of transmission interruption. This will help consolidate the gains achieved through the existing national deworming programs, while maximizing the impact of donated drugs. Against this backdrop, our study evaluates a novel community-wide deworming strategy designed to account for hookworm transmission dynamics. Although the low baseline prevalence limits direct generalizability to higher-intensity communities, our findings provide valuable insights into assessing the feasibility of STH transmission interruption in low-intensity settings.*

*We have now added a paragraph to this effect in our Discussion section, which read as follows (page 20, lines 416-427):*

*The baseline prevalence of MHI infections in the study villages (<1.2%) was below the WHO target for STH elimination as a public health problem (<2% prevalence of MHI infections) (48). Although this low baseline prevalence limits direct generalizability to higher-intensity communities, the findings of this study provide valuable insights for assessing the feasibility of STH transmission interruption in low-intensity settings. It is important to note that the WHO target of <2% prevalence of MHI infections is aimed at morbidity control rather than transmission interruption. It is estimated that interrupting STH transmission in endemic communities will require a post-treatment STH prevalence of ≤2% (Truscott et al. Parasit Vectors. 2017: 321.). However, this threshold may be difficult to attain through targeted deworming of PSAC and SAC alone (Roy et al. PLoS Negl Trop Dis. 2013;7(2):e2027), particularly in hookworm-endemic areas where untreated adult reservoirs of infection sustain high reinfection rates (Jia et al. PLoS Negl Trop Dis. 2012; 6: e1621). Therefore, alternative deworming strategies need to be explored if transmission interruption is to be achieved.”*

2. Details are needed on the selection and participation in the coprological survey. Please clarify how the random subset of participants were selected? Were they selected at the household level? At the community level? Any stratification by age? What proportion of the community were selected? Were participants from each community recruited proportional to population?

**RESPONSE:** *Participants for the coprological surveys were randomly selected from among the permanent residents of the study villages, aged 2–70 years (both years inclusive), who met the eligibility criteria. Individuals were excluded if they had syndromic or serological evidence of HIV infection or other immunocompromising conditions, were pregnant, had a history of seizure, epilepsy or other known neurological disorders, or had a known hypersensitivity to albendazole. At study initiation (baseline), a door-to-door survey of all households in the study villages was conducted to collect socio-demographic data (age, sex, education, occupation, and household socio-economic status), which served as the sampling frame for the cross-sectional coprological surveys. For each survey round (baseline; after every MDA cycle; and 3-, 6-, 9-, 12- and 24-month follow-up), the eligible residents were randomly ordered, and the first 30 individuals, per village, who provided written informed consent and at least one stool sample, were enrolled.*

*The Methods section has now been revised to clarify the participant selection and sampling procedure for the coprological surveys, which reads as follows (page 08, lines 168-175):*

*“Participants for the coprological surveys were randomly selected from among the permanent residents of the study villages aged 2-70 years (both years inclusive) who met the eligibility criteria; exclusions included syndromic or serological evidence of HIV infection or other immunocompromising conditions, pregnancy, a history of seizures, epilepsy or other neurological disorders, and known hypersensitivity to albendazole. The door-to-door baseline census provided the sampling frame. For each survey round, eligible residents were randomly ordered, with the first 30 participants from each village, who provided written informed consent and at least one stool sample, enrolled.”*

3. Please clarify the timing of the follow up in the three sets of treatment villages. Was the pre-intervention synchronized across the three treatments? And then three-month post-intervention was staggered across the three treatments (i.e. 3 months after single cycle, three months after end of second cycle (one month after 1st treatment group), and three months after end of fourth cycle (8 months after the single cycle group)? Any seasonal impacts on transmission in the study communities?

**RESPONSE:** *The detailed sampling plan for the cross-sectional coprological surveys is presented in Figure 2 of the protocol paper (Sarkar et al. Contemp Clin Trials Commun. 2017; 5: 49-55). For operational efficiency, villages assigned to the four-cycle intervention were enrolled first, followed by those assigned to the two- and one-cycle interventions. However, all post-treatment assessments were standardized relative to the timing of the final mass drug administration (MDA) round, within each intervention arm. In each study village, a baseline coprological survey was conducted to estimate hookworm prevalence and infection intensity, with up to three stool samples collected per participant. Albendazole was then administered to all eligible residents according to the assigned mass drug administration (MDA) schedule (one, two or four rounds). Post-MDA surveys were conducted 2-4 weeks after each MDA round. Follow-up surveys were conducted at 3, 6, 9, 12, and 24 months after the final MDA round for all intervention arms.* *Specifically, follow-up sampling began three months after the single MDA round in one-cycle villages, three months after the second round in two-cycle villages (i.e., four months after baseline), and three months after the fourth round in four-cycle villages (i.e., eight months after baseline), ensuring comparable follow-up intervals across all arms. This has now been clarified in the Methods section of the manuscript, which reads as follows (page 08, lines 160-166):*

*“Cross-sectional coprological surveys were conducted at baseline, after every MDA cycle, and every three months post-MDA, for one year, and at the end of two years to ascertain the prevalence and intensity of hookworm infection. The follow-up surveys were standardized relative to the timing of the final MDA round within each intervention arm, beginning three months after the single MDA round in one-cycle villages, three months after the second round in two-cycle villages (i.e., four months after baseline), and three months after the fourth round in four-cycle villages (i.e., eight months after baseline) (Figure 2 of (16)*)*”.*

*The study design did not allow for ascertainment of seasonality of hookworm transmission in the study area.*

4. Please provide demographic details on the participants in the coprological surveys across the timepoints. These could be included as a supplemental file; but important to see the characteristics of the participants on whom the prevalence and intensity data are based.

**RESPONSE:** *The demographic profile of the participants who provided one or more stool samples in the coprological surveys (baseline, and at 3, 6, 9, 12 and 24 months of follow-up) have now been included as a supplementary figure (Figure S2). We have also inserted a sentence in the Results section to explain the demographic profile of non-participants in the coprological surveys, which reads as follows (page 13, lines 267-270):*

*“The individuals who missed MDAs were more likely to be teenagers (15-19 years) or adult males (20-54 years), reflecting the migratory pattern of the study population. These demographic subgroups (teenagers and adult males) were also less likely to participate in the coprological surveys (Figure S2).”*

5. Discussion:
   a. Line 354-357 – Results conclude no statistically significant difference between three different treatment groups 24 months after intervention (lines 318-320). Lines 354-357 assert significantly lower prevalence and intensity after four cycles. Please integrate the nuance here that is repeated in the next paragraph, of the difference no longer being apparent at 24 months.

**RESPONSE:** *We have modified the sentence to integrate the nuance that the difference in prevalence and intensity of hookworm infection observed up to one-year post-treatment was not visible at two years. The sentence now reads (page 19, lines 370-374):*

*“Two additional cycles of MDA (one month apart) after 6 months (i.e., a total of four-MDA cycles), however, resulted in a significantly lower prevalence and intensity of hookworm infection up to one year post-treatment, which likely reflects the benefits of multiple rounds of MDA in endemic communities, although the gains were not visible after two years.”*

b. Consider including the limitation that the criteria for WHO elimination as a public health problem were met prior to onset of the study.

**RESPONSE:** *The fact that the baseline prevalence of MHI infections (<1.2%) in the study villages was already below the WHO threshold for elimination as a public health problem (<2% prevalence of MHI infections) has now been mentioned in the Discussion section, and its potential impact on the generalizability of the study findings to settings with high infection intensity highlighted. The statement reads as follows (page 20, lines 416-420):*

*“The baseline prevalence of MHI infections in the study villages (<1.2%) was below the WHO target for STH elimination as a public health problem (<2% prevalence of MHI infections) (48). Although this low baseline prevalence limits direct generalizability to higher-intensity communities, the findings of this study provide valuable insights for assessing the feasibility of STH transmission interruption in low-intensity settings.”*

6. Minor issues:
   a. Line 68 – ‘along-with’ doesn’t need a hyphen.

**RESPONSE:** *The hyphen has been removed, as suggested. The sentence now reads (page 04, lines 66-69):*

*“To control STH-associated morbidity, the World Health Organization (WHO) advocates targeted periodic deworming of pre-SAC, SAC and women of child-bearing age living in endemic areas as the mainstay STH-control strategy, along with provision of adequate sanitation, improved hygiene practices and access to safe water (5).”*

b. Line 75-76 – please clarify what the appropriate reference is. Findings from large-scale community intervention trials are referenced in citations 11 and 12, but 12 is a global modeling study, not a large-scale community intervention trial.

**RESPONSE:** *The citation # 12 was incorrect. We have now updated it to: Pullan RL, et al. Effects, equity, and cost of school-based and community-wide treatment strategies for soil-transmitted helminths in Kenya: a cluster-randomised controlled trial. Lancet. 2019; 393(10185): 2039-2050.*

**Additional Editor Comments:**

The manuscript has been reviewed and I concur with the issues raised. Overall this is a well designed and implemented study. However, there are questions raised by the reviewer about the methods and results that when addressed will provide greater clarity. In addition, a revised manuscript should address the "overarching question" raised by the reviewer regarding the low prevalence of moderate to high intensity infections in the study population. The authors should explain how this affects interpretation of the findings of the study and implications for extrapolating results to other endemic communities.

**RESPONSE:** *Thank you for your thoughtful comment. We have incorporated a paragraph into the Discussion section (page 20, lines 430-441) highlighting the fact that the baseline prevalence of MHI infections in the study villages (<1.2%) was below the WHO threshold for defining STH elimination as a public health problem (<2%). We have also elaborated on the implications of this finding, particularly with respect to achieving transmission interruption in low-intensity settings, consolidating the gains of national STH control programs, and maximizing the impact of donated drugs. In addition, we highlight the limited generalizability of these findings to high-intensity settings.*

**Journal Requirements:**

1. Please ensure that the CRediT author contributions listed for every co-author are completed accurately and in full.

At this stage, the following Authors/Authors require contributions: Rohan Michael Ramesh, Rajiv Sarkar, Vasanthakumar Velusamy, Srinivasan Venugopal, Anuradha Rose, Venkata R. Mohan, Vinohar Balraj, Vedantam Rajshekhar, Kuryan George, Jayaprakash Muliyil, Nicholas C. Grassly, Simon J. Brooker, Roy M. Anderson, Gagandeep Kang, and Sitara Swarna Rao Ajjampur. Please ensure that the full contributions of each author are acknowledged in the "Add/Edit/Remove Authors" section of our submission form.

The list of CRediT author contributions may be found here:

<https://journals.plos.org/plosntds/s/authorship#loc-author-contributions>

**RESPONSE:** *Thank you! The CRediT author contributions listed for all co-authors have now been added, as requested.*

2. Tables should not be uploaded as individual files. Please remove these files and include the Tables in your manuscript file as editable, cell-based objects. For more information about how to format tables, see our guidelines: https://journals.plos.org/plosntds/s/tables

**RESPONSE:** *As requested, the tables are now included within the manuscript file as editable, cell-based objects.*
